# The *N*-glycan Glycoprotein Deglycosylation Complex (Gpd) from *Capnocytophaga canimorsus* Deglycosylates Human IgG

**DOI:** 10.1371/journal.ppat.1002118

**Published:** 2011-06-30

**Authors:** Francesco Renzi, Pablo Manfredi, Manuela Mally, Suzette Moes, Paul Jenö, Guy R. Cornelis

**Affiliations:** Biozentrum der Universität Basel, Basel, Switzerland; University of California San Diego, United States of America

## Abstract

*C. canimorsus 5* has the capacity to grow at the expenses of glycan moieties from host cells *N*-glycoproteins. Here, we show that *C. canimorsus 5* also has the capacity to deglycosylate human IgG and we analyze the deglycosylation mechanism. We show that deglycosylation is achieved by a large complex spanning the outer membrane and consisting of the Gpd proteins and sialidase SiaC. GpdD, -G, -E and -F are surface-exposed outer membrane lipoproteins. GpdDEF could contribute to the binding of glycoproteins at the bacterial surface while GpdG is a endo-β-*N*-acetylglucosaminidase cleaving the *N*-linked oligosaccharide after the first *N*-linked GlcNAc residue. GpdC, resembling a TonB-dependent OM transporter is presumed to import the oligosaccharide into the periplasm after its cleavage from the glycoprotein. The terminal sialic acid residue of the oligosaccharide is then removed by SiaC, a periplasm-exposed lipoprotein in direct contact with GpdC. Finally, most likely degradation of the oligosaccharide proceeds sequentially from the desialylated non reducing end by the action of periplasmic exoglycosidases, including β-galactosidases, β-*N*-Acetylhexosaminidases and α-mannosidases.

## Author Summary

*Capnocytophaga canimorsus* are Gram-negative bacteria from the normal oral flora of dogs and cats. They cause rare but severe infections in humans that have been bitten or simply licked by a dog or cat. Fulminant septicemia and peripheral gangrene with a high mortality are the most common symptoms. A surprising feature of these bacteria is their capacity to feed by foraging the glycan moieties of glycoproteins from animal cells, including phagocytes. Here we show that *C. canimorsus* can also deglycosylate human IgGs reinforcing the idea that this property of harvesting host glycoproteins may contribute to pathogenesis. We also unravel the complete deglycosylation system which belongs to a large family of systems devoted to foraging complex glycans, found exclusively in the *Capnocytophaga-Flavobacteria-Bacteroides* group, and whose archetype is the starch harvesting system Sus. It is the first system devoted to deglycosylation of glycoproteins to be characterized.

## Introduction

*Capnocytophaga* are capnophilic Gram negative bacteria that belong to the family of *Flavobacteriaceae* in the phylum *Bacteroidetes* and colonize the oral cavity of diverse mammals including humans [1], [2]. *Capnocytophaga canimorsus*, a usual member of dog's mouths flora [3], [4], was discovered in 1976 [5] in patients that underwent dramatic infections after having been bitten, scratched or simply licked by a dog. These infections occur, worldwide, with an approximate frequency of one per million inhabitants per year. They generally begin with flu symptoms and evolve in a few days into fulminant septicaemia and peripheral gangrene with a mortality as high as 40% [5], [6], [7], [8], [9]. A few recent observations help understanding the high aggressiveness of *C. canimorsus* for humans. First, *C. canimorsus* are able to escape complement killing and phagocytosis by human polymorphonuclear leukocytes (PMN's) [10], [11]. They also escape detection and phagocytosis by macrophages, which results in a lack of release of pro-inflammatory cytokines [12]. In addition to this passive evasion from innate immunity, 60% of the strains are able to block the killing of *Escherichia coli* phagocytosed by macrophages [3], [10] and some strains even block the onset of pro-inflammatory signalling induced by an *E. coli* lipopolysaccharide (LPS) stimulus [12]. The molecular bases of these immunosuppressive mechanisms are not understood yet. However, their study led to the serendipitous discovery that the fastidious *C. canimorsus* grow readily upon direct contact with mammalian cells including phagocytes. This property was found to be dependent on a sialidase (SiaC) allowing *C. canimorsus* to harvest amino sugars of glycan chains from host cell glycoproteins [13]. Recently, we reported the complete 2,571,405-bp genome sequence and the surface proteome of strain *Cc5* (Manfredi *et al.*, submitted). Among others, this study unravelled the existence of 13 complex feeding systems encoded by polysaccharide utilization loci (PULs), a hallmark of the *Cytophaga-Flavobacteria-Bacteroides* (CFB) group [14], [15]. The archetype of these systems is the Sus system, pioneered by the laboratory of A. Salyers and allowing *Bacteroides thetaiotaomicron* to forage starch. It is composed of the surface-exposed SusCDEF protein complex [15], [16] and the SusAB periplasmic proteins [17]. SusC resembles a TonB-dependent transporter essential for energy-dependent import of starch oligosaccharides into the periplasm [18] while SusD is a α-helical starch-binding lipoprotein [19], [20]. SusE and SusF are other surface-exposed lipoproteins that reinforce starch binding [17]. Finally, the outer membrane α-amylase SusG hydrolyses surface-bound starch [19]. *B. thetaiotaomicron* has 88 of these PULs, identified essentially by the presence of a pair of adjacent *susC*-like and *susD*-like alleles. Interestingly, expression of some PULs is upregulated in the presence of mucin *O*-glycans or glycosaminoglycans (GAGs), indicating that *B. thetaiotaomicron* also forages on host glycans, primarily the *O*-glycosylated mucin [14] but these glycoprotein foraging systems have not been characterized so far. Although *Streptococcus oralis*, a firmicute from the human oral flora and *S. pneumoniae* have been shown to remove and metabolize *N*-linked complex glycans of human glycoproteins [21], [22], [23], no Sus-like-*N*-linked glycan foraging system has been described in detail. One of the 13 Sus-like systems from *C. canimorsus 5* is involved in the capacity to grow on mammalian cells and to deglycosylate glycoproteins. It is encoded by chromosome locus *PUL5* and accounts for 12% of the *Cc5* surface proteins. Since it contributes to survival in mice. it can be seen as a new type of bacterial virulence factor (Manfredi *et al.*, submitted). Here, we present a detailed molecular characterization of this *PUL5*-encoded foraging complex and we show that it deglycosylates human immunoglobulins G (IgG). Among others, we show that it cleaves *N*-linked glycan moieties between two GlcNAc residues and we show its functional relation with sialidase.

## Results

### Genetic analysis of the *PUL5* locus

**Figure 1:**
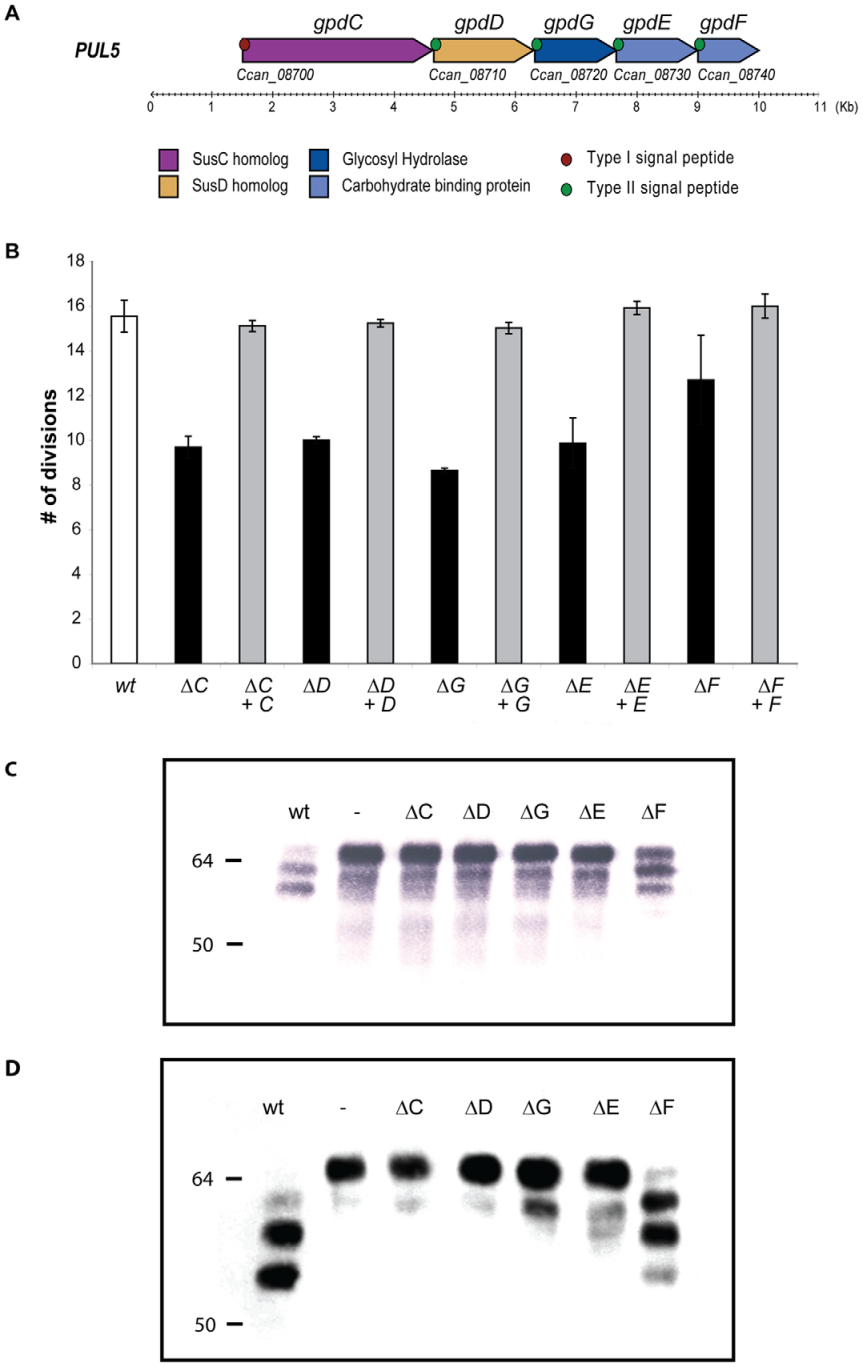
Genetic analysis of the *PUL5* locus. (A). Schematic representation of the *PUL5* putative operon (top: new gene designation; below: gene codes derived from the annotation of the genome (Manfredi *et al.* submitted). (B). Growth of the various individual *gpd* knockout (black) and complemented (grey) mutants on HEK293 cells (moi = 0.2; 23 hours growth). (C). Glycosylation state of fetuin samples incubated for 2 hours in the presence of the different strains, monitored by staining with SNA that recognizes terminal sialic acid (2–6) linked to Gal or to GalNAc. (D). Western blot analysis with anti-fetuin antibodies of fetuin samples incubated as in (C).

*PUL5* consists of the five genes *Ccan_08700*–*Ccan_08740*. *Ccan_08700* encodes a SusC-like integral outer membrane (OM) protein presumably forming a pore in the OM while *Ccan_08710* is a SusD-like protein presumably involved in substrate binding [20]. Since the locus was shown to confer the capacity to deglycosylate proteins (Manfredi *et al.*, submitted), we named the five genes *gpd* (for glycoprotein deglycosylation) and we called *gpdC* and *gpdD* the genes encoding homologs to SusC and SusD, respectively. The five *gpd* genes seem to be organized as an operon in the order *gpdC*, *gpdD*, *gpdG*, *gpdE* and *gpdF* (**Fig. 1A**). GpdG is predicted to be an endo-β-*N*-acetylglucosaminidase and GpdE has similarities with the Concanavalin A-like lectins/glucanases superfamily on its 108 C-terminal amino acids and could have a substrate-binding role analogous to that of GpdD. Finally, GpdF shows homology to the galactose-binding domain-like superfamily on its 136 C-terminal amino acids suggesting again a role in glycan binding.

In order to investigate what is the function of the individual Gpd proteins we constructed single *gpd* genes knockout strains. None of the knockout mutants was significantly affected in its growth on blood agar plates. In contrast, deletion of any of the *gpdC, -D, -G or -E genes* led to a clear reduction of growth on HEK293 cells while deletion of *gpdF* had only a slight effect (**Fig. 1B**). Complementation of the deleted genes with plasmid-borne genes expressed from the natural *gpdC* promoter completely restored growth to the wildtype (wt) level indicating that none of the mutation was polar.

In order to determine whether the reduced growth of the mutants was due to a defect in protein deglycosylation, we incubated wt *Cc5* bacteria and the *gpd* mutant bacteria with fetal calf serum protein fetuin, taken as a standard glycoprotein. Fetuin contains 3 *O*-linked glycans (accounting for 20% of fetuin-bond carbohydrates) and 3 *N*-linked glycans (80% of fetuin-bond carbohydrates) [24]. We monitored glycosylation by staining with *Sambucus nigra* agglutinin (SNA), a lectin that recognizes terminal sialic acids on glycans. As shown in **Fig. 1C**, fetuin that had been incubated with wt *Cc5* reacted much less with SNA and appeared as two, still sialylated smaller degradation products. As shown by Manfredi *et al.* (submitted), this indicated that partial deglycosylation had occurred and progressed further than a simple desialylation. In contrast, fetuin that was incubated with the *gpdC*, *-D*, *-G* and *-E* mutant bacteria was unaffected, indicating that no desialylation occurred in the absence of these *gpd* genes, although sialidase SiaC [13] was not directly affected. Fetuin incubated with the *gpdF* mutant showed a slight desialylation indicating that fetuin deglycosylation was not completely abolished as with the other mutants. Fetuin glycosylation was also monitored by immuno-blotting with anti-fetuin antibodies. As shown in **Fig. 1D**, the size of fetuin was shifted down after incubation with wt *Cc5* bacteria while the protein migration rate was unchanged after incubation with the *gpdC*, *-D*, *-G* and *-E* mutant bacteria. After incubation with *gpdF* mutant bacteria, fetuin did undergo a size shift but not as marked as when incubated with wt bacteria. Taken together these results indicate that partial fetuin deglycosylation was strictly dependent on the activity of proteins GpdC, -D, -G, -E and, to a lesser extend -F. Finally, our data strongly suggest that the defect in growth of the *gpd* mutants onto HEK293 cells was due to a defect in the ability to deglycosylate host glycoproteins.

### GpdG is an endo-β-*N*-acetylglucosaminidase

**Figure 2:**
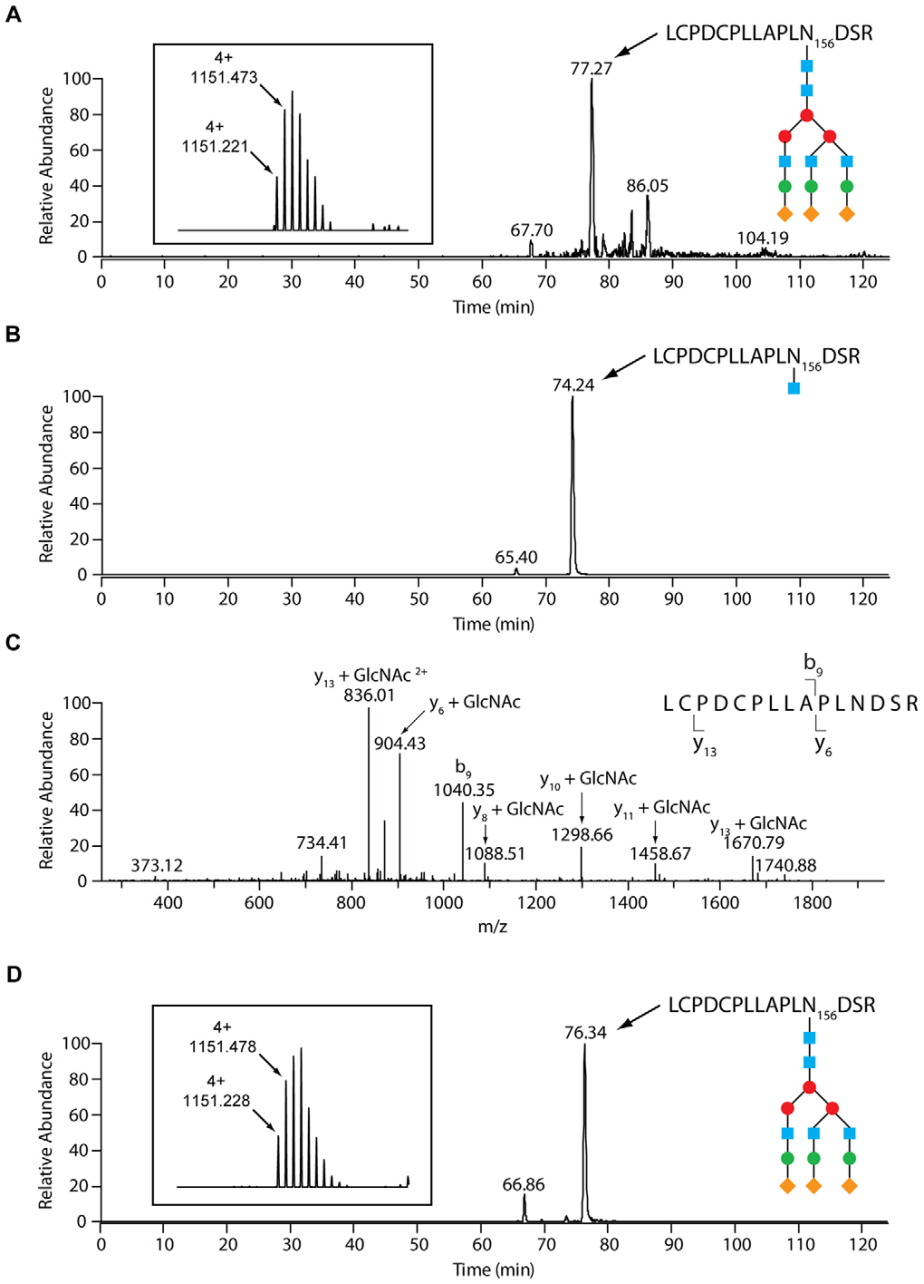
LC-MS analysis reveals an endo-β-*N*-acetylglucosaminidase activity of GpdG. Glycosylation analysis of fetal calf serum fetuin. (A) N156 glycosylation of untreated bovine fetuin. Selected ion chromatogram for the quadruply charged tryptic peptide whose m/z is in accordance with a GlcNAc_5_Man_3_Gal_3_Sial_3_ glycosyl moiety on the LCPDCPLLAPLNDSR peptide. The inset shows the isotope pattern for the N156 glycopeptide. The blue squares represent GlcNAc, the red and green circles Man and Gal, respectively, and the orange diamonds Sial. (B) Selected ion chromatogram for the doubly charged N156 GlcNAc-modified LCPDCPLLAPLNDSR glycopeptide of fetuin that had been incubated with wild-type *Cc5*. The sugar moieties are designated as in (A). (C) Fragmentation spectrum of the N156- GlcNAc species with the y- and b-ions that conclusively show the GlcNAc modification of N156. (D) N156 glycosylation of bovine fetuin that had been treated with the *ΔgpdG* strain. Selected ion chromatogram for the quadruply charged tryptic peptide carrying the presumed GlcNAc_5_Man_3_Gal_3_Sial_3_ glycosyl moiety on the LCPDCPLLAPLNDSR peptide. The inset shows the isotope pattern for the N156 glycopeptide. The sugar moieties are designated as in (A).

GpdG is annotated as an endo-β-*N*-acetylglucosaminidase (Manfredi *et al.*, submitted), *i.e* an endo-glycosidase that cleaves *N*-linked glycan structures at the base of the glycan in between two GlcNAc molecules. Hence, it should leave one GlcNac molecule attached to the protein. Fetuin is reported to be glycosylated on the three asparagine residues N99, N156 and N176 [24]. Analysis by liquid chromatography-mass spectrometry (LC-MS) of trypsin-digested fetuin showed that the main glycosylation site resides on N156. The m/z is in accordance with the peptide LCPDCPLLAPLNDSR carrying the GlcNAc_5_Man_3_Gal_3_Sial_3_ sugar (**Fig. 2A**). N176 was found to carry a sugar with a Hex6HexNAc5NeuAc4 composition, but its site occupancy was much lower than N156. Only trace amounts of glycans were found attached to N99. After incubation of fetuin with wt *Cc5* bacteria, LC-MS analysis revealed the presence of a peptide whose mass indicated that only one GlcNAc moiety remained linked to N156 (**Fig. 2B**). The fragmentation spectrum of this peptide fully confirmed the presence of the GlcNAc moiety on N156 (**Fig. 2C**). Due to the low site occupancy of N176, deglycosylation of N176 to the GlcNAc moiety was too weak to be detected. The conversion of GlcNAc_5_Man_3_Gal_3_Sial_3_ to GlcNAc on N156 suggests an endo-β-*N*-acetylglucosaminidase dependent deglycosylation.

To confirm that fetuin deglycosylation was due to the Gpd complex activity and in particular to the GpdG glycosyl hydrolase activity, we then analysed fetuin after incubation with the *gpdG* knockout bacteria. Fetuin incubated in the presence of these mutant bacteria turned out to remain fully glycosylated (**Fig. 2D**) indicating that no cleavage occurred in the absence of the enzyme.

**Figure 3:**
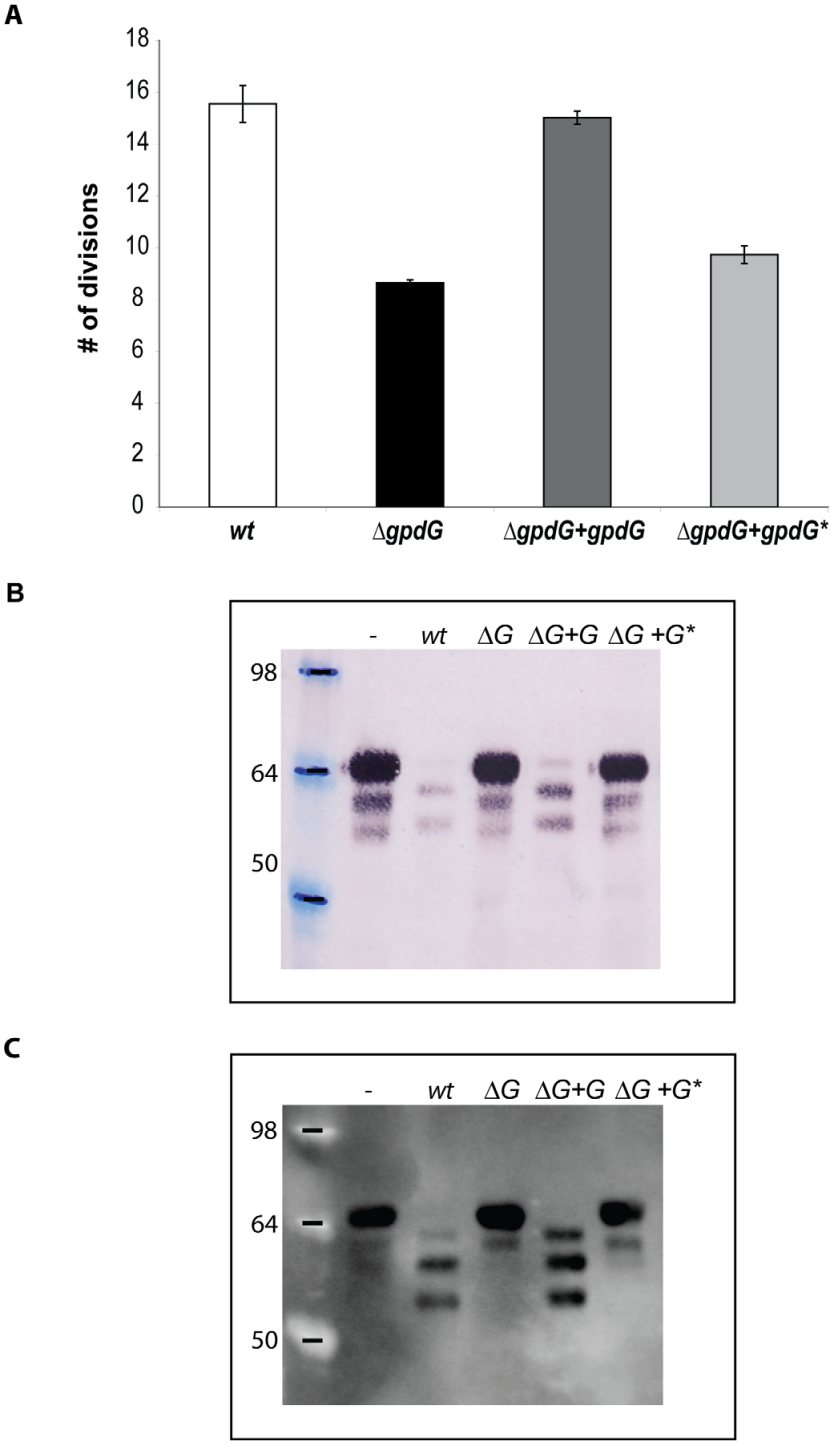
The F_197_DGFDIDWE_205_ chitinase motif of GpdG is the catalytic site. E_205_ from GpdG was substituted with a glycine. (A): Number of divisions after 23 h growth on HEK293 cells of the *ΔgpdG* mutant complemented with gpdG* encoding the catalytic mutant. (B): Fetuin glycosylation state of samples incubated for 3 hours in the presence of the different strains, determined by staining with the *Sambucus nigra* lectin (SNA) that recognizes terminal sialic acid (2–6) linked to Gal or to GalNAc. (C): same as B after western blot analysis with anti-fetuin antibodies.

The sequence of GpdG was then compared to those of two endo-β-*N*-acetylglucosaminidases, namely EndoS from *Streptococcus pyogenes* capable of deglycosylating *N*-linked glycans from the γ chain of human immunoglobulins [25], and EndoF from *Flavobacterium meningosepticum* capable of cleaving off high-mannose and complex glycan *N*-linked from several glycoproteins including immunoglobulins [26]. It appeared that a chitinase motif present in these two enzymes was conserved in GpdG (FDGFDIDWE). In order to further confirm the endo-β-*N*-acetylglucosaminidase activity of GpdG we substituted the essential E205 residue [26] with a glycine and tested the growth on HEK293 cells of the *gpdG* mutant strain expressing in trans the GpdG catalytic mutant. As shown in **Fig. 3A**, the GpdG catalytic mutant was impaired in growth. We then tested the fetuin deglycosylation ability of the GpdG catalytic mutant. As shown by the lectin staining in **Fig. 3B** and by the immuno-blotting in **Fig. 3C**, bacteria endowed with the GpdG catalytic mutant were completely impaired in fetuin deglycosylation. We conclude from all these experiments that GpdG is an endo-β-*N*-acetylglucosaminidase.

### The Gpd complex deglycosylates human IgG

**Figure 4:**
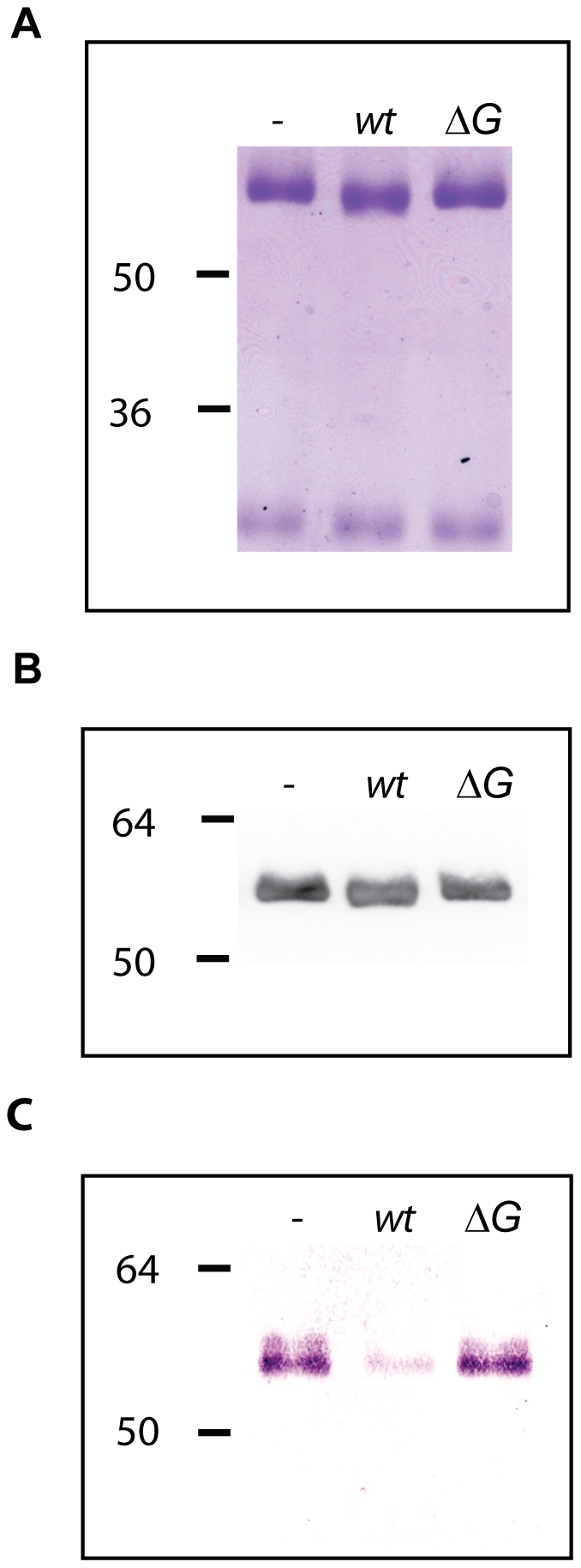
Human IgG deglycosylation. Glycosylation state of human IgG samples incubated for 3 hours in the presence of wt and *ΔgpdG* bacteria monitored by Coomassie staining (A), western blot analysis with anti-IgG antibodies (B) and staining with SNA (C).

**Figure 5:**
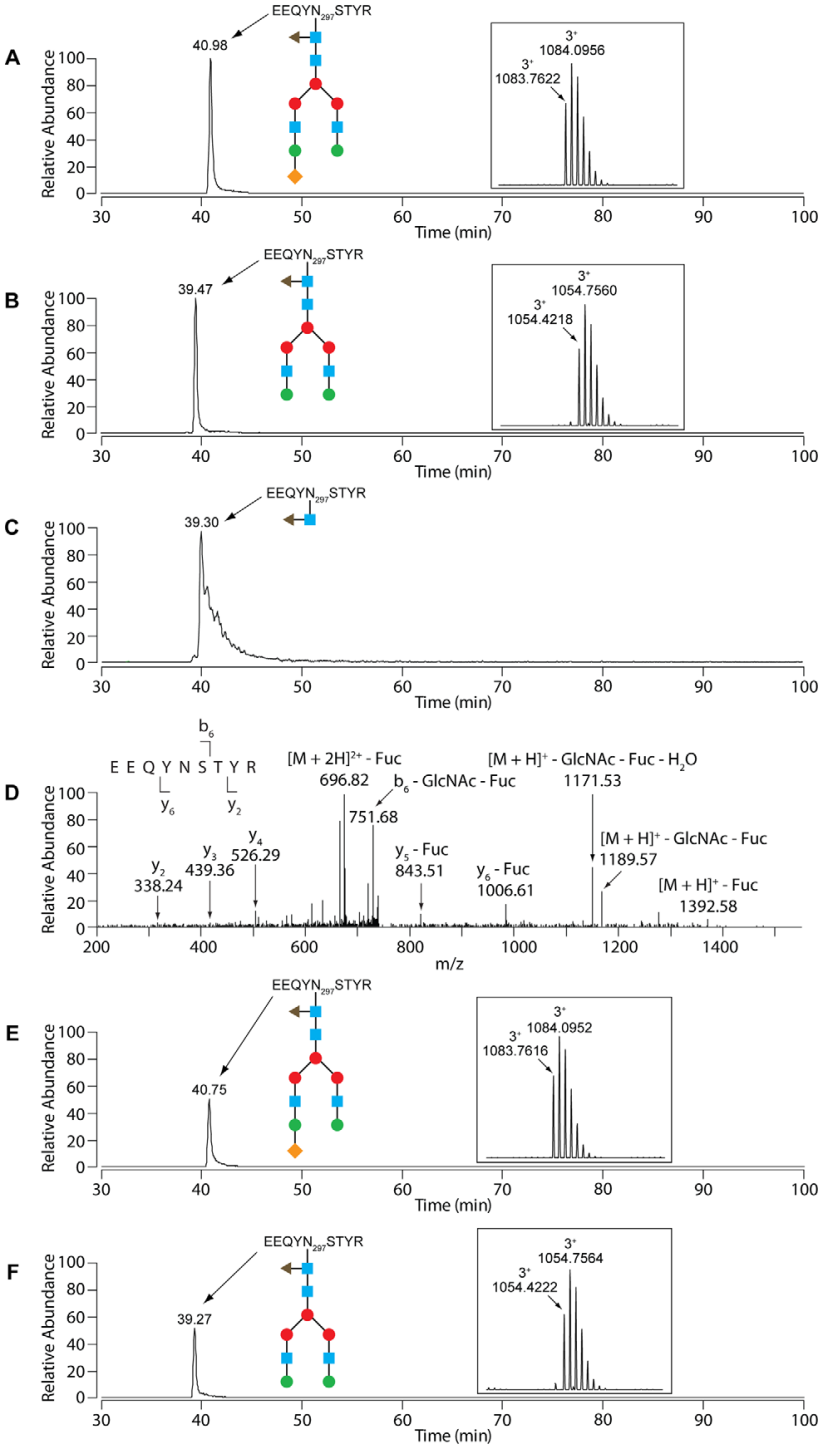
LC-MS analysis of IgG. Glycosylation analysis of human IgG. (A) N297 glycosylation of untreated human IgG. Selected ion chromatogram for the triply charged tryptic peptide whose m/z is in accordance with a GlcNAc_4_Man_3_Fuc_1_Gal_2_Sial_1_ glycosyl moiety on the EEQYNSTYR peptide. The inset shows the isotope pattern for the N297 glycopeptide. The blue squares represent GlcNAc, the red and green circles Man and Gal, respectively, the orange diamonds Sial, and triangle Fuc. (B) N297 glycosylation of untreated human IgG. Selected ion chromatogram for the triply charged tryptic peptide whose m/z is in accordance with the GlcNAc_4_Man_3_Fuc_1_Gal_2_ glycosyl moiety on the EEQYNSTYR peptide. The inset shows the isotope pattern for the N297 glycopeptide. The sugar moieties are designated as in (A). (C) Selected ion chromatogram for the doubly charged N297 GlcNAc_1_Fuc_1_-modified glycopeptide of IgG that had been incubated with wild-type *Cc5*. The sugar moieties are designated as in (A). (D) Fragmentation spectrum of the N297 GlcNAc_1_Fuc_1_ species with the y- and b-ions that conclusively show the GlcNAc_1_Fuc_1_ modification of N297. (E) N297 glycosylation of human IgG that had been treated with the *ΔgpdG* strain: selected ion chromatogram for the triply charged tryptic peptide carrying the presumed GlcNAc_4_Man_3_Fuc_1_Gal_2_Sial_1_ glycosyl moiety on the EEQYNSTYR peptide. The inset shows the isotope pattern for the N297 glycopeptide. For the sugar structure see (A). (F) N297 glycosylation of human IgG that had been treated with the *ΔgpdG* strain: selected ion chromatogram for the triply charged tryptic peptide carrying the presumed GlcNAc_4_Man_3_Fuc_1_Gal_2_ glycosyl moiety on the EEQYNSTYR peptide. The inset shows the isotope pattern for the N297 glycopeptide. For the sugar structure see (A).

Since GpdG has the same chitinase motif as EndoF and EndoS, known to deglycosylate *N*-linked glycans from the γ chain of human IgGs [25], [26], we tested whether the Gpd complex would also be able to deglycosylate the heavy chain of IgGs. Cleavage of the N297-linked glycan moiety by EndoS was shown to determine a size shift of ∼3 KDa [25]. After incubation of purified human IgG with wt *Cc5* bacteria, the molecular mass of the γ chain underwent a slight size shift (**Fig. 4A and B**) while the mass of the light chains was unchanged (**Fig. 4A**). In contrast incubation with *ΔgpdG* knockout bacteria did not alter the γ chain size indicating that the cleavage was GpdG dependent. To confirm that the size reduction of the γ chain was due to the removal of the glycan moiety, IgG was stained with SNA. As shown in **Fig. 4C**, the SNA signal of the γ chain was significantly reduced after incubation with wt *Cc5*. In contrast the γ chains remained fully glycosylated after incubation with *ΔgpdG* bacteria. Analysis by LC-MS of trypsin-digested IgG showed that N297 from this sample of IgG mainly bears a GlcNAc_4_Man_3_Gal_2_Sial_1_Fuc_1_ chain or a GlcNAc_4_Man_3_Gal_2_Fuc_1_ chain (**Fig. 5A–B**). After incubation of this sample of IgG with wt *Cc5* bacteria, LC-MS analysis revealed the presence of a peptide whose mass indicated that only a GlcNAc_1_Fuc_1_ moiety remained linked to N297 (**Fig. 5C**). The fragmentation spectrum of this peptide fully confirmed the presence of the GlcNAc_1_Fuc_1_ moiety on N297 (**Fig. 5D**). LC-MS analysis of IgG after incubation with *ΔgpdG* bacteria revealed exactly the same profile as in the untreated sample indicating that no deglycosylation has occurred (**Fig. 5E–F**). The conversion of GlcNAc_4_Man_3_Gal_2_Sial_1_Fuc_1_ and GlcNAc_4_Man_3_Gal_2_Fuc_1_ to GlcNAc_1_Fuc_1_ on N297 indicates an endo-β-*N*-acetylglucosaminidase (GpdG) dependent deglycosylation. These data indicated that, like *F. meningosepticum* and *S. pyogenes*, *C. canimorsus* has the capacity to deglycosylate IgGs.

### GpdD, -G, -E and -F are lipoproteins and lipid modification is fundamental for the complex activity

**Figure 6:**
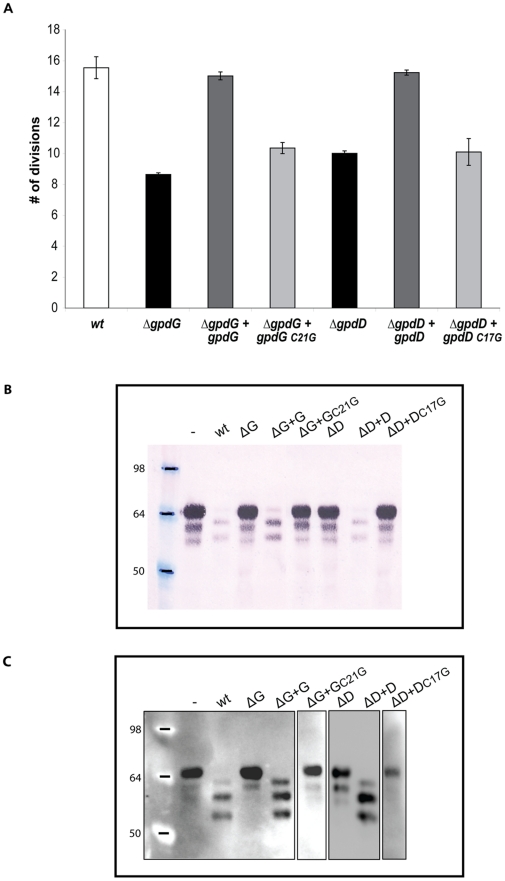
Lipid modification of GpdD and GpdG is essential for their activity. (A) Number of divisions after 23 h growth on HEK293 cells of the *ΔgpdG* bacteria complemented with *gpdD_C17G_* and *gpdG_C21G_*. (B) Fetuin glycosylation state of samples incubated for 2 hours in the presence of the different strains, determined by staining with SNA. (C) Same as B analyzed by western blot with anti-fetuin antibodies.

The GpdD, -G, -E and –F proteins belong to the OM and surface proteomes of *Cc5* (Manfredi *et al.*, submitted). In addition, these proteins are endowed with a signal peptidase II consensus signal peptide. Altogether, this suggests that they could be lipoproteins anchored to the outer leaflet of the outer membrane and exposed at the surface of the bacterium (Manfredi *et al.*, submitted). In order to determine whether the lipidation of the Gpd proteins is required for their function, we generated soluble periplasmic versions of GpdD and GpdG by substituting the cystein residue of the lipobox with a glycine. We then tested the ability of the periplasmic variants of GpdD and GpdG to complement the growth deficiency of the *gpdD* and *gpdG* knockout strains on HEK293 cells. As shown in **Fig. 6**, both the GpdD and GpdG periplasmic variant were unable to complement the growth deficiency indicating that lipid modification is necessary for the proper localization and function of the proteins. This conclusion was reinforced by the fact that bacteria endowed with periplasmic GpdD or GpdG were unable to deglycosylate fetuin (**Fig. 6**). Hence, we infer that GpdD and GpdG are lipoproteins that are anchored in the outer leaflet of the outer membrane and exposed to the bacterial surface. The same presumably applies to GpdE and GpdF since they have also a lipobox and they are also part of the surface proteome (Manfredi *et al.*, submitted).

### The Gpd proteins form a deglycosylation complex associated with sialidase

**Figure 7:**
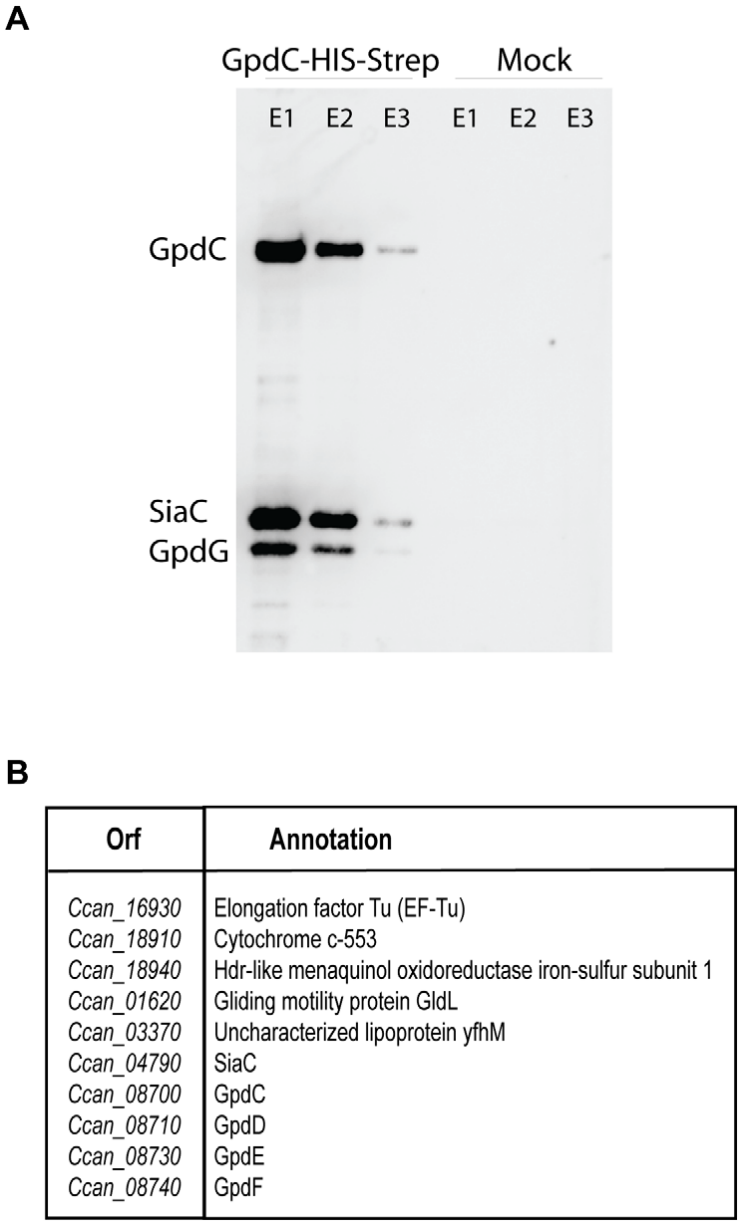
Gpd proteins form a complex with sialidase. Streptavidine affinity purification of GpdC-His-Strep expressed from its natural promoter in a *ΔgpdC background*. (A) Detection by western blot of GpdC (anti-His antibody), GpdG (anti-GpdG) and Sialidase (anti-SiaC) in the elution fractions. (B) List of protein identified by Mass spectrometry in the elution fractions.

In order to assay whether the five Gpd proteins interact with each other to form a complex at the bacterial surface, we performed a two-step affinity purification with a His-Strep tagged version of GpdC. Analysis by immuno-blot and mass spectrometry (**Fig. 7**) of the purified fraction revealed the presence, together with GpdC, of GpdD, -G, -E and –F, indicating a stable interaction between all these proteins. Furthermore, six other proteins, among which SiaC (**Fig. 7**), co-purified with the complex.

### Sialidase is a periplasmic lipoprotein that interacts with GpdC

SiaC has been previously shown [13] to be essential to sustain growth of *Cc5* in the presence of eukaryotic cells due to its role in the glycoprotein deglycosylation process. We thus focused our attention on the sialidase-Gpd complex interaction. The co-purification of SiaC with GpdC strongly suggested that SiaC is associated to the Gpd complex, although it is encoded far away from *PUL5*. However, unlike the Gpd proteins, sialidase was not identified in the surface proteome of *Cc5* (Manfredi *et al.*, submitted). On the other hand, earlier immunofluorescence assays suggested that sialidase is localized on the bacterial surface and removal of the signal sequence of sialidase prevented growth on cells [13]. In order to better understand the interplay between SiaC and Gpd proteins in the glycoprotein deglycosylation process, we decided to clarify its localization.

**Figure 8:**
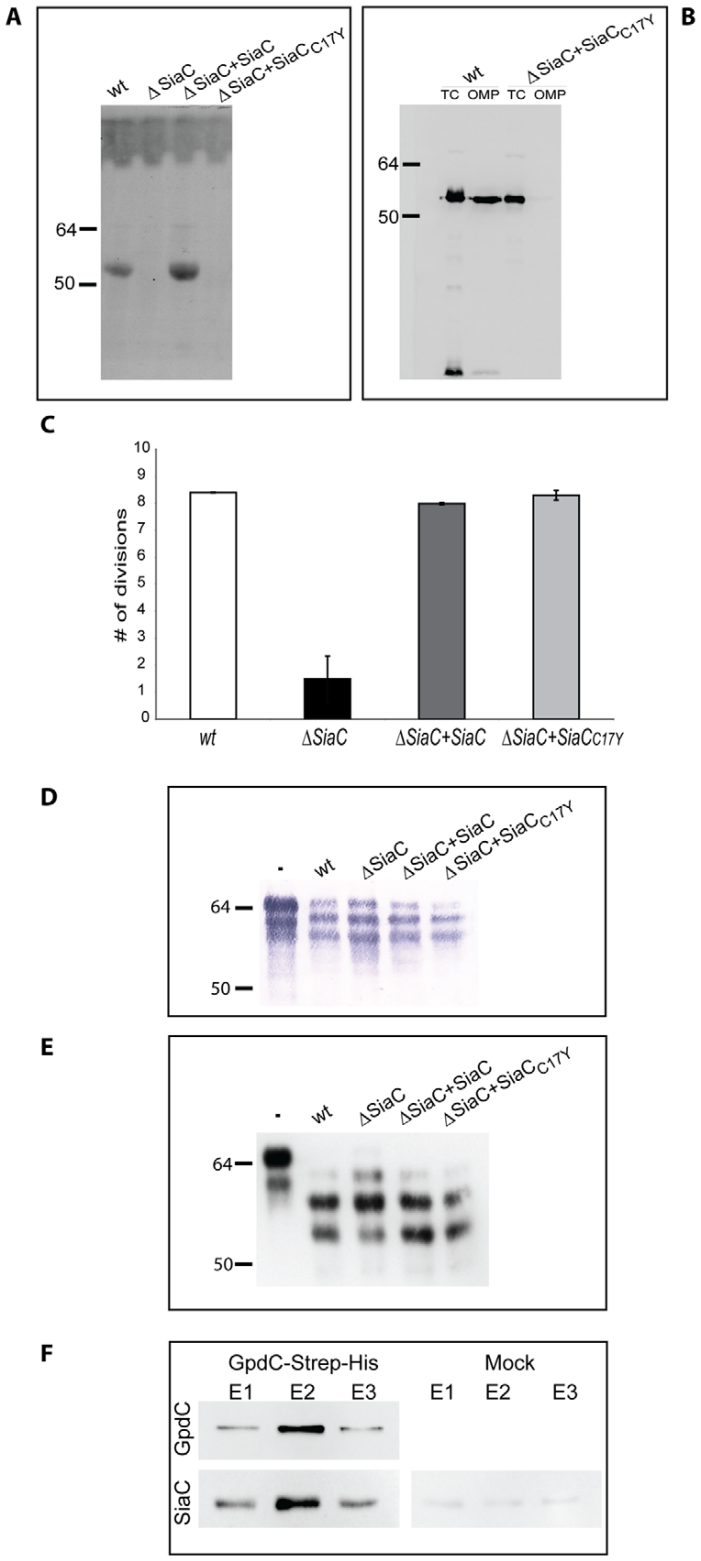
Sialidase localization and interaction with GpdC. (A) Autoradiography of ^3^H-palmitate labeled sialidase in different bacteria. (B) Detection of sialidase by western blot analysis (anti-SiaC antibody) in total cell extracts (TC) and outer membrane protein (OMP) fractions of *Cc5* wt and *ΔsiaC* bacteria complemented with the soluble periplasmic sialidase (SiaC_C17Y_). (C) Number of divisions after 23 hours growth on HEK293 cells of *ΔsiaC* bacteria expressing SiaC or SiaC_C17Y_. (D) Fetuin glycosylation state after 2 hours of incubation in the presence of the different strains, determined by staining SNA. (E) Same as D, analyzed by western blot with anti-fetuin antibodies. (F) Co-purification of SiaC with GpdC-Strep-His produced in a *ΔgpdCDGE* background. GpdC was detected with anti-Strep antibody and SiaC with anti-SiaC antibodies.

Since the sialidase sequence analysis revealed the presence of a signal peptide with a lipobox in the N-terminal sequence, we first sought to determine whether SiaC is a lipoprotein. We incubated *Cc5* and sialidase mutant (*siaC*) bacteria encoding SiaC_C17Y_ in the presence of tritiated palmitate and analyzed the total proteins by SDS-PAGE and fluorography (**Fig. 8A**). Sialidase appeared indeed to be lipidated and the C17Y mutation completely prevented this lipid modification. The analysis of outer membrane proteins isolated by sarcosyl extraction confirmed that sialidase but not its C17Y variant was associated with the OM (**Fig. 8B**). We conclude from these experiments that SiaC is a lipoprotein anchored into the outer membrane.

In order to define whether it is exposed towards the outside like GpdDGEF or towards the periplasm, we tested whether the periplasmic Sia_C17Y_ could restore the growth deficiency of the *siaC* mutant strain. In contrast to what was observed for GpdD and GpdG, expression of Sia_C17Y_ in trans did fully restore the growth defect (**Fig. 8C**) indicating that the localization of sialidase in the periplasm and the absence of association with the outer membrane did not prevent its function. This data pointed to the direction of a periplasmic localization of SiaC rather than a surface-exposed localization as was previously suggested [13].

The association between sialidase and the Gpd complex obviously suggests that the two work cooperatively. This was already suggested by the fact that the *gpd* mutant bacteria did not remove the terminal sialic acid residues from fetuin, although SiaC was functional in these mutants (**Fig. 1C**). We then tested the ability of the *siaC* knockout bacteria to deglycosylate fetuin. SNA lectin staining (**Fig. 8D**) and immuno-blotting (**Fig. 8E**) clearly showed the same fetuin deglycosylation pattern for the wt and *siaC* mutant bacteria. These results indicate that the endo-cleavage of fetuin *N*-glycans, operated by the Gpd complex is completely independent from the activity of SiaC. However, the evidence that SiaC activity is essential for growth on Hek293 cells (**Fig. 8C**), suggests that removal of the glycan terminal sialic acid is nevertheless a crucial step for the subsequent glycan catabolism process. This indicates that the Gpd complex acts upstream of SiaC. Since the Gpd complex includes the GpdC porin-like protein, this sequential order is perfectly compatible with a periplasmic localization of sialidase. Sialic acid removal would thus occur in the periplasm after the glycan has been cleaved off and transported through the GpdC OM channel.

If this model was correct, the interaction between the periplasmic SiaC and the GpdC complex could only occur through a direct interaction with GpdC, since the other Gpd proteins are surface exposed. To test this prediction, we expressed a C-terminally Strep-His double tagged GpdC in a *gpdCDGE* multi knockout strain and we performed a two-step affinity purification of GpdC. The analysis by immuno-blotting (**Fig. 8F**) of the fractions eluted after the second purification step showed that SiaC did indeed co-purify with GpdC indicating that SiaC and GpdC do indeed interact directly with each other. The complete deglycosylation complex would thus consist of the surface-exposed lipoproteins GpdDGEF and the periplasm-exposed lipoprotein SiaC, all of them associated to the porin-like GpdC (**Fig. 8**).

## Discussion

**Figure 9:**
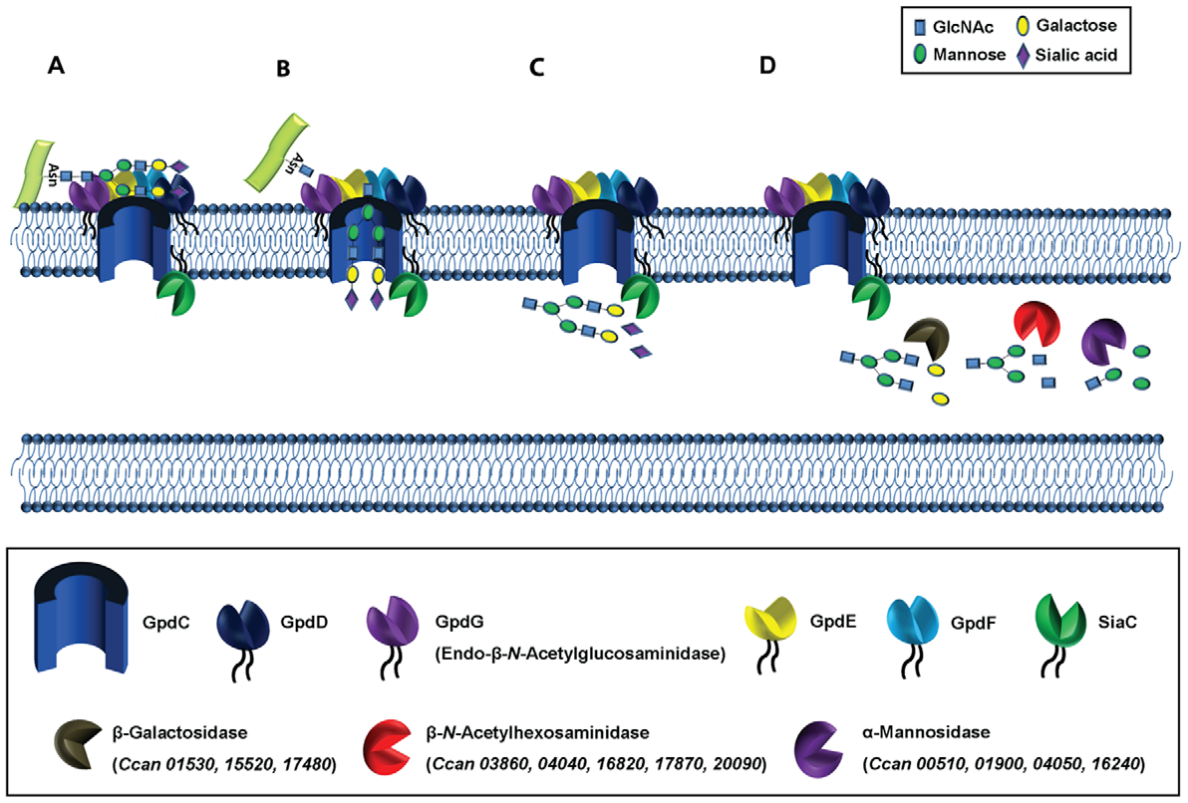
Functional model of complex *N*-linked glycan moieties deglycosylation processing by *C. canimorsus*. Individual glycan processing steps are illustrated. (A) The glycan moiety is bound at the bacterial surface by the Gpd complex. (B) The glycan mopiety is endo-cleaved by GpdG and imported into the periplasm trough the GpdC pore. (C) Terminal sialic acid is cleaved by sialidase (SiaC). (D) The glycan is further processed by the sequencial activity of several periplasmic exoglycosidases.

Our previous work has shown that *C. canimorsus* deglycosylates surface glycoproteins from the host and sustains its growth on the glycan moieties [13]. Here, we showed that this deglycosylating activity is achieved by the joined action of the *PUL5*-encoded Gpd complex and sialidase [13]. PUL5 consists of the five *gpdCDGEF* genes. GpdC, an homolog of the archetypal SusC [17], likely represents the specific OM porin of the system. GpdD is an homolog of SusD, a starch-binding protein [16], [20] and hence most likely a glycoprotein-binding protein. On the basis of their annotation, we propose that GpdE and GpdF are also glycan-binding proteins. GpdG was annotated as an endo-β-*N*-acetylglucosaminidase (Manfredi *et al.*, submitted) and this annotation was shown to be correct. Indeed mass spectrometry analyses demonstrated that GpdG deglycosylates the tribranched GlcNAc_5_Man_3_Gal_3_Sial_3_ glycan structure linked to N156 from the model glycoprotein fetuin, leaving one GlcNac residue on the protein. GpdDGEF were predicted to be lipoproteins (Manfredi *et al.*, submitted). Replacement of the critical cysteine of the lipoprotein signal peptide from GpdD and GpdG completely abolished the deglycosylating activity, indicating that a periplasmic location did not sustain the activity. These data, together with the fact that the two proteins belong to the surface proteome indicate that these two lipoproteins are exposed to the surface and not to the periplasm. We assume the same is true for GpdE and F since, like GpdD, they are thought to bind glycans, they contain a lipobox and they belong to the surface proteome. Interestingly, all the five Gpd proteins could be co-purified with the porin-like GpdC, indicating that they all form one single complex at the bacterial surface. Unexpectedly, not only GpdD, -G, -E and -F co-purified with GpdC but also SiaC. Although SiaC was known to be part of the catabolic process, SiaC is not encoded together with GpdCDGEF (Manfredi *et al.*, submitted) and it was not anticipated that the interaction would be so close. SiaC turned out to be also a lipoprotein but, unlike GpdD and GpdG, it was still functional when it was directed to the periplasm, unlipidated. We inferred from this observation that, contrary to our initial report, SiaC is a periplasm-oriented lipoprotein. Thus, the observations presented here suggest the model illustrated in **Fig. 9**: The surface-exposed GpdCDEF complex captures the *N*-linked complex glycan moieties of glycoproteins, which are then detached from the protein by GpdG and internalized by GpdC. As soon as they reach the periplasm, SiaC removes the terminal sialic acid. This sequence of events is strongly supported by the observation that *gpd* mutant bacteria do not desialylate fetuin, although SiaC is functional in these mutants (**Fig. 1**). After desialylation, the oligosaccharide would be sequentially degraded by periplasmic exoglycosidases and the monosaccharides would be transferred to the cytosol. This last step of the model is supported by the fact that the genome encodes three putative β-galactosidases (*Ccan_01530*, *Ccan_15520*, *Ccan*_*17480*), five putative β-*N*-acetylhexosaminidases (*Ccan_03860*, *Ccan*_*04040*, *Ccan_16820*, *Ccan*_*17870*, *Ccan_20090*) and four putative α-mannosidases (*Ccan*_*00510*, *Ccan*_*01900*, *Ccan_04050* and *Ccan*_*16220*), all of them endowed with a signal peptide I or II, and none of them surface exposed (Manfredi *et al.*, submitted). The β-galactosidase and α-mannosidase activities were confirmed in the crude extract (data not shown). The three β-galactosidases seemed actually redundant since they could all be individually knocked out without affecting the growth on cells (data not shown).

This global model strikingly reminds the archetypal Sus system shown to consist of one single complex made of SusCDEF [16]. It is thought that SusG, an *endo*-acting enzyme, generates internal cuts in a bound starch molecule and releases oligosaccharides larger than maltotriose, which are then transported by SusC into the periplasmic compartment. In the periplasm, glycoside hydrolases SusA and SusB then degrade the oligosaccharides into their component sugars prior to final transport to the cytosol [27], [28]. The two systems are thus remarkably conserved, although they adapted to different complex saccharides.

To our knowledge, the Gpd system is the first Sus-like system devoted to foraging *N*-linked glycoproteins. It contributes to sustain growth of *C. canimorsus* at the expenses of cultured cells (Manfredi *et al.*, submitted). Since *C. canimorsus* has 13 PULs (Manfredi *et al.*, submitted), it is very likely that some of them could be devoted to the harvest of *O*-linked glycans, but this activity has not been identified thus far. The best approach would probably be to look for upregulation in the presence of *O*-linked glycoproteins, as was done in *B. thetaiotaomicron* [29]. Deglycosylation of *N*-linked glycans is not unprecedented among pathogens and commensals. As mentioned earlier, two *streptococci*, *S. pyogenes* and *S. oralis* have this remarkable property. In the case of *S. pyogenes*, this activity is exerted towards IgGs by secreted endoglycosidase EndoS and it does not seem to play a major role in nutrient acquisition [25]. In contrast, in *S. oralis*, the activity was shown to sustain growth [30]. It is interesting to notice that *S. oralis*, like *C. canimorsus*, is emerging as an important opportunistic pathogen originating from the oral flora. This commonality between two very different bacteria from the same ecosystem suggests first that the capacity to deglycosylate host proteins is a favourable trait in the mouth ecosystem and, second, could favour opportunistic infections. Deglycosylation of IgGs is very likely to contribute to a generalized infection as discussed by Collin and Olsen [25] but, for *C. canimorsus*, one cannot exclude that deglycosylation of other host proteins would also significantly contribute to pathogenesis.

Our data demonstrate that PUL-encoded lipoproteins are surface-exposed. Prolipoproteins are exported through the Sec pathway and then acylated at the periplasmic leaflet of the inner membrane (IM) by the sequential action of glyceryl transferase, O-acyl transferase(s) and prolipoprotein signal peptidase (signal peptidase II). A mature lipoprotein harbours as a first aminoacid a cysteine residue that is lipid modified with a N-Acyl diacyl Glyceryl group which serves to anchor the protein to the IM. In Gram-negative bacteria some lipoproteins are destined for the OM. These proteins are extracted from the IM, transported across the periplasm and inserted in the inner leaflet of the OM by the Lol pathway (for review see refs [31], [32]. Insertion of lipoproteins into the outer leaflet of the OM is however established in some pathogens like *Borrelia* but the pathway is neither well documented nor well understood [32]. Since bacteria from the *Cytophaga-Flavobacteria-Bacteroides* group massively insert lipoproteins in the outer leaflet of the OM, we postulate that they have an original system dedicated to the transport of lipoproteins across the OM but this system still needs to be identified and investigated.

## Materials and Methods

### Bacterial strains and growth conditions

#### Conventional bacterial growth conditions and selective agents

The strains used in this study are listed in Table 1. *Escherichia coli* strains were routinely grown in LB broth at 37°C. *C. canimorsus* bacteria were routinely grown on heart infusion agar (Difco) supplemented with 5% sheep blood (Oxoid) for 2 days at 37°C in the presence of 5% CO_2_. To select for plasmids, antibiotics were added at the following concentrations: 10 µg/ml erythromycin (Em), 10 µg/ml cefoxitin (Cf), 20 µg/ml gentamicin (Gm), 100 µg/ml ampicillin (Ap) and 50 µg/ml kanamycin (Km).

**Table 1:** Bacterial strains used in this study.

Bacterial strains	Description or genotype	Reference or source
***E. coli***		
S17-1	*hsdR17 recA1* RP4-2-*tet::*Mu1*kan::*Tn7; Sm^r^	[34]
***C. canimorsus***		
*Cc5*	Human fatal septicemia after dog bite 1995	[12]
*Cc5ΔsiaC*	Replacement of *Ccan_00790* by *ermF*; Em^r^	[13]
*Cc5ΔPUL5*	Replacement of *Ccan_08700*, *Ccan_08710*, *Ccan_08720*, *Ccan_08730* by *ermF* : Em^r^	Manfredi *et al.*, submitted
*Cc5ΔgpdC*	Replacement of *Ccan_08700* by *ermF* using primers 5073, 5074, 5075, 5083; Em^r^	This study
*Cc5Δ gpdD*	Replacement of *Ccan_08710* by *ermF* using primers 4850, 4851, 4854, 4855; Em^r^	This study
*Cc5ΔgpdG*	Replacement of *Ccan_08720* by *ermF* using primers 5001, 5002, 5005, 5006; Em^r^	This study
*Cc5ΔgpdE*	Replacement of *Ccan_08730* by *ermF* using primers 5951, 5952, 5953, 5954; Em^r^	This study
*Cc5ΔgpdF*	Replacement of *Ccan_08740* by *ermF* using primers 5955, 5956, 5957, 5958; Em^r^	This study

#### Growth of *Cc5* bacteria on HEK293 cultured cells

Human Embryonic Kidney 293 cells (HEK293) were cultured in DMEM (Invitrogen) with 10% (v/v) fetal calf serum (Invitrogen) and 1 mM sodium pyruvate. Cells were grown in medium without antibiotics in a humidified atmosphere enriched with 5% CO_2_ at 37°C. Bacteria were harvested by gently scraping colonies off the agar surface and resuspended in PBS. A total of 4×10^4^ bacteria were incubated with 2×10^5^ HEK293 cells (MOI = 0.2) in a final volume of 1 ml medium devoid of antibiotics for 23 h.

### Mutagenesis and allelic exchange

Mutagenesis of *Cc5* wt has been performed has described in ref [33] with slight modifications. Briefly, replacement cassettes with flanking regions spanning approximately 500 bp homologous to direct *gpd* framing regions were constructed with a three-fragment overlapping-PCR strategy. First, two PCRs were performed on 100 ng of of *Cc5* genomic DNA with primers A and B (**Table 2**) for the upstream flanking regions and with primers C and D for the downstream regions. Primers B and C contained an additional 5′ 20-nucleotide extension homologous to the resistance *ermF* insertion cassette. The *ermF* resistance cassette was amplified from plasmid pMM106 DNA with primers 5502 and 5503. All three PCR products were cleaned and then mixed in equal amounts for PCR using Phusion polymerase (Finnzymes). The initial denaturation was at 98°C for 2 min, followed by 12 cycles without primers to allow annealing and elongation of the overlapping fragments (98°C for 30 s, 50°C for 40 s, and 72°C for 2 min). After the addition of external primers (A and D), the program was continued with 20 cycles (98°C for 30 s, 50°C for 40 s, and 72°C for 2 min 30 s) and finally 10 min at 72°C. Final PCR products consisted in *gpd::ermF* insertion cassettes and were then digested with PstI and SpeI for cloning into the appropriate sites of the *C. canimorsus* suicide vector pMM25. Resulting plasmids were transferred by RP4-mediated conjugative DNA transfer from *E. coli* S17-1 to *C. canimorsus 5* to allow integration of the insertion cassette. Transconjugants were then selected for presence of the *ermF* cassette, checked for sensitivity to cefoxitin and the deleted regions were sequenced.

**Table 2:** Oligonucleotides used in this study.

Ref.	Name	Sequence 5′-3′	Restriction	Gene	PCR
5502	ermF-fw	CTACGAAGGATGAAATTTTTCAGGGACAAC		*ermF*	
5503	ermF-rev	AACAGTGCTTTTATCTACTCCGATAGCTTC		*ermF*	
5081	PgpdC-fw	CGATGTCGACTGAATATGTTGTACATTTGTG	SalI		
5469	PgpdC-rev	CATACCATGGCAATAATAAAATGAATTAG	NcoI		
5082	gpdC-rev	CCACTAGTACCTATAATGAAGCTTTAATTGC	SpeI	*gpdC*	
5467	gpdC-His-rev	TGACTAGTTAATGATGATGATGATGATGAGCACCACCAGCACCACCTAATGAAGCTTTAATTGCAATACC	SpeI	*gpdC*	
5530	gpdC-Strep-rev	TGACTAGTTATTTTTCAAATTGAGGATGTGACCAAGCTCCTCCAGCTCCTCCATGATGATGATGATGATGAGC	SpeI	*gpdC*	
5001	gpdGKO-1.1-fw	CGCTGCAGGATTGTAATACCCATCTTTG	PstI	*gpdG*	A
5002	gpdGKO-1.2-rev	GAGTAGATAAAAGCACTGTTGAGACTTGATAACAAGTAAA		*gpdG*	B
5005	gpdGKO-2.1-fw	AAAAATTTCATCCTTCGTAGTTACTTTGATAAGTATATTA		*gpdG*	C
5006	gpdGKO-2.2-rev	CCACTAGTCTGACGCCAAATTAGAGTCA	SpeI	*gpdG*	D
4850	gpdDKO-1.1-fw	CCCTGCAGTTAATAAGAAATGAAAAAATAC	PstI	*gpdD*	A
4851	gpdDKO-1.2-rev	GAGTAGATAAAAGCACTGTTAATACGGTAAGGGACCAAAC		*gpdD*	B
4854	gpdDKO-2.1-fw	AAAAATTTCATCCTTCGTAGTTCTGAAAATGGGGTAAGCA		*gpdD*	C
4855	gpdDKO-2.2-rev	CCACTAGTAAGATTATCTTGTATTAGGATTC	SpeI	*gpdD*	D
5073	gpdCKO-1.1-fw	CCCTGCAGACTTATAGCTCTTGCGTGCGGACTTTGG	PstI	*gpdC*	A
5074	gpdCKO-1.2-rev	GAGTAGATAAAAGCACTGTTGCACTTCGTTGAATGTTAATGCCAGCCA		*gpdC*	B
5075	gpdCKO-2.1-fw	AAAAATTTCATCCTTCGTAGTGAAGGCGGTTCAATGACAGCAGTG		*gpdC*	C
5083	gpdCKO-2.2-rev	CCACTAGTATTCGGGATCAAAAGGCGCTGACAA	SpeI	*gpdC*	D
5951	gpdEKO-1.1-fw	GGCTGCAGCGGTTACCATCCACAAGAGAAAG	PstI	*gpdE*	A
5952	gpdEKO-1.2-rev	GTTGTCCCTGAAAAATTTCATCCTTCGTAGAATTTACTATTTTTTAGGTAATCTG		*gpdE*	B
5953	gpdEKO-2.1-fw	GAAGCTATCGGAGTAGATAAAAGCACTGTTGATTTCCTAATGTTGATTTTAATACC		*gpdE*	C
5954	gpdEKO-2.2-rev	GCACTAGTGGGTGAGACATCAGATACTTG	SpeI	*gpdE*	D
5955	gpdFKO-1.1-fw	GGCTGCAGGTTTGAAGCAGCGGGTACTAATCC	PstI	*gpdF*	A
5956	gpdFKO-1.2-rev	GTTGTCCCTGAAAAATTTCATCCTTCGTAGCCCTACCAGTAATACTGTTGTGAG		*gpdF*	B
5957	gpdFKO-2.1-fw	GAAGCTATCGGAGTAGATAAAAGCACTGTTGGGAGGAGATCAATATGTTGATATAAATG		*gpdF*	C
5958	gpdFKO-2.2-rev	GCACTAGTCGGCTTTTTCGAATGAAACGAAC	SpeI	*gpdF*	D
6133	gpdD-fw	CATACCATGGGAAAAAAATACTTTATGATAGGTGCTTTATCTTTAGC	NcoI	*gpdD*	
6057	gpdD-rev	GCTCTAGATTATCTTGTATTAGGATTCACATCCCACC	XbaI	*gpdD*	
5008	gpdG-fw	CATGCCATGGGAAAAAAAAATATTATAAAATGGGG	NcoI	*gpdG*	
6055	gpdG-rev	GCTCTAGACTATTTTTTAGGTAATCTGATAATTAATTGCTC	XbaI	*gpdG*	
5959	gpdE-fw	CATACCATGGGAAAGAAATTACATATCTTATTTGTTATCG	NcoI	*gpdE*	
5960	gpdE-rev	GCTCTAGATTAAAATTCTACTTTGGTATTAAAATC	XbaI	*gpdE*	
5962	gpdF-fw	CATACCATGGGAAAAAAACATATAAAAATTTTATTTCTCACAACAG	NcoI	*gpdF*	
5963	gpdF-rev	GCTCTAGACTAATAAAATTCTAATTCATTTATATCAAC	XbaI	*gpdF*	
6056	gpdDCys-fw	CATACCATGGGAAAAAAATACTTTATGATAGGTGCTTTATCTTTAGCTACAATTTCTGGTACGAAAG	NcoI	*gpdD*	
6054	gpdGCys-fw	CATACCATGGGAAAAAAAAATATTATAAAATGGGGTTTAGCAATACTTATAGGGGTAGCTTCTGTAA	NcoI	*gpdG*	
6060	gpdG-E/G-fw	CCAAAAGATATTGACTGGGGACCTACTGTGGGTAATCATGGAAG		*gpdG*	
6061	gpdG-E/G-rev	CTTCCATGATTACCCACAGTAGGTCCCCAGTCAATATCTTTTGG		*gpdG*	
5045	siaCCys-fw	CTTTTGTCGGCTTATGGAAGCCAAAAA		*siaC*	
5046	siaCCys-rev	TTTTTGGCTTCCATAAGCCGACAAAAG		*siaC*	

### Construction of complementation and expression plasmids

Plasmid pPM1, used for complementation and expression of the Gpd proteins, is a derivative of the *E. coli*- *C. canimorsus* shuttle vector pMM47A.1 [33]. pMM47A.1 *ermF* promoter region was cleaved with *Sal*I and *Nco*I and the 117 nucteotides upstream the *gpd*C starting codon sequence, containing the putative *gpdC* promoter, was cloned using the same restriction sites. Full length *gpdC*, -*D*, -*G*, -*E* and -*F* were amplified with the specific primers listed in **Table 3** and cloned into plasmid pPM1 into *Nco*I and *Xba*I restriction sites leading to the insertion of a glycine at position 2.

**Table 3:** Plasmids used in this study.

Plasmid	Description	Reference or source
pMM47.A	*ori*_ColE1_, *ori*_pCC7_, Ap^r^ ,Cf^r^, *E. coli - C. canimorsus* expression shuttle vector.	[33]
pPM1	pMM47.A where the *ermF* promoter has been replaced by the stronger *gpd* promoter: 117 bp upstream of the *gpdC* ORF start codon were amplified with primers 5081 and 5469 and cloned into pMM47.A using *Sal*I and *Nco*I restriction sites.	This study
pPM2	Full length *gpdC* containing its putative promoter region amplified with primers 5081 and 5082 and cloned into pMM47.A using *Sal*I and *Spe*I restriction sites.	This study
pPM3	Full length *gpdC* with a C-terminal His-Strep double tag amplified by 2-step overlapping PCR with primers 5081, 5467 and 5530 and cloned into pMM47.A using *Sal*I and *Spe*I restriction sites.	This study
pFR4	Full length *gpdD* amplified with primers 6133 and 6057 and cloned in pPM1 using *Nco*I and *Xba*I restriction sites.	This study
pFR5	Full length *gpdG* amplified with primers 5008 and 6055 and cloned in pPM1 using *Nco*I and *Xba*I restriction sites.	This study
pFR6	Full length *gpdE* amplified with primers 5959 and 5060 and cloned in pPM1 using *Nco*I and *Xba*I restriction sites.	This study
pFR7	Full length *gpdF* amplified with primers 5062 and 5063 and cloned in pPM1 using *Nco*I and *Xba*I restriction sites.	This study
pFR8	Full length *gpdD* with a C17G point mutation amplified with primers 6056 and 6057 and cloned in pPM1 using *Nco*I and *Xba*I restriction sites.	This study
pFR9	Full length *gpdG* with a C21G point mutation amplified with primers 6054 and 6055 and cloned in pPM1 using *Nco*I and *Xba*I restriction sites.	This study
pFR10	Full length *gpdG* with a E205G point mutation amplified by overlapping PCR using primers 5008/6061 and 6060/6055 and cloned in pPM1 using *Nco*I and *Xba*I restriction sites.	This study
pMM121.1	Full length *siaC* amplified by inverse PCR using primers 5045+5046 on pMM52 as a template to insert to C17Y substitution in *siaC*.	This study
pMM25	*ori*_ColE1_, Km^r^, Cf^r^.Suicide vector for *C. canimorsus*.	[33]
pMM52	Full length *siaC* with a C-terminal His tag cloned in pMM47.A using *Nco*I and *Xba*I restriction sites.	[13]
pMM106	*ori*_ColE1_ , Km^r^ , Cf^r^ , Ery^R^ , Mutator plasmid for the replacement of *siaC* by *ermF*	[33]

The E205G substitution inactivating the catalytic site of GpdG was introduced by site directed mutagenesis by overlapping PCR using primers 5008/6061 and 6060/6055 and cloned in pPM1 using *Nco*I and *Xba*I restriction sites leading to plasmid pFR10 (gpdG*). The C17G substitution of GpdD was introduced by site directed mutagenesis amplifying by PCR using primers 6056 and 6057 and cloning *Nco*I/*Xba*I in pPM1 leading to plasmid pFR8.

The C21G substitution of GpdG was introduced by site directed mutagenesis amplifying by PCR using primers 6054 and 6055 and cloning *Nco*I/*Xba*I in pPM1 leading to plasmid pFR9.

The C17Y substitution of SiaC was introduced by site directed mutagenesis amplifying by inverse PCR using primers 5045 and 5046 using as pMM52 as template leading to plasmid pMM121.1.

C-terminal His-Strep double tagged *gpdC* was amplified by two-step overlapping PCR using primers 5081, 5467 and 5530 and cloned in pMM47.A using *Sal*I and *Spe*I restriction sites leading to plasmid pPM3.

### Fetuin deglycosylation analyses and lectin stainings

Bacteria were collected from blood agar plates and resuspended in PBS at OD_600_ = 1. 100 µl of bacterial suspensions were then incubated with 100 µl of a fetal calf serum fetuin (Sigma F2379) solution (0.1 g.l^−1^) for 120 minutes at 37°C. As negative control, 200 µl of 1∶2 diluted fetuin solution alone was incubated for 120 minutes at 37°C. Samples were then centrifuged for 5 min at 13000× g, supernatant collected and loaded in a 12% SDS gel. Samples were analyzed by immunoblotting (Fetuin, Rabbit anti-Bovine RIA, UCBA699/R1H, ACCURATE CHEMICAL & SCIENTIFIC CORPORATION) and lectin stainings were performed with Sambucus nigra lectin (SNA) according to manufacturer recommendations (DIG Glycan Differentiation Kit, 11210238001, Roche).

### Human IgG deglycosylation analyses and lectin stainings

Bacteria were collected from blood agar plates and resuspended in PBS at OD_600_ = 1. 100 µl of bacterial suspensions were then incubated with 100 µl of a purified human IgG (Invitrogen, 02-7102) solution (0.5 g.l^−1^) for 180 minutes at 37°C. As negative control, 200 µl of 1∶2 diluted IgG solution alone was incubated for 180 minutes at 37°C. Samples were then centrufiged for 5 min at 13000× g, supernatant collected and and loaded in a 12% SDS gel. Samples were analyzed by Coomassie blue staining, immunoblotting [Goat Anti-Human IgG (Fc specific)-FITC antibody, F9512 Sigma] and lectin stainings were performed with SNA according to manufacturer recommendations (DIG Glycan Differentiation Kit, 11210238001, Roche).

### Fetuin and IgG mass spectrometric analyses

Fetuin (Sigma F2379) and human IgG (Invitrogen, 02-7102) were reduced with 10 mM TCEP at 37°C for 1 hour and alkylated with 50 mM iodoacetamide for 15 min at room temperature. Fetuin and IgG were digested with trypsin at an enzyme to protein ratio of 1∶50 (w/w) at 37°C overnight. The peptides were desalted on C18 StageTips (Thermo Fisher Scientific, Reinach, Switzerland) according to the manufacurer's recommendations. The fetuin and IgG peptides were analysed on an LTQ Orbitrap instrument (Thermo Fisher, San José, CA, USA) coupled to an Agilent 1200 nano pump according to (Manfredi *et al.* submitted).

### Outer membrane protein purification

Bacteria were collected from blood agar plates and resuspended in 3 ml ice cold HEPES 10 mM (pH 7.4) at OD_600_ = 1. Bacterial suspensions were then sonicated on ice until they turned clear and spun at 15600× g for 2 minutes at 4°C. Supernatants were transferred and centrifuged again for 30 minutes at 15600× g at 4°C. Pellets were resuspended in 2 ml HEPES 10 mM with 1% sarcosyl (N-Lauroylsarcosine sodium salt, Sigma) and incubated at room temperature for 30 minutes. Finally, samples were centrifuged at 15600 g for 30 min at 4°C and pellet resuspended in 0.1 ml HEPES. Samples were checked for quality and quantity on silver stained SDS-PAGE and analysed by MS/MS.

### Gpd proteins and sialidase co-purification

*Cc5* Δ*gpdC* bacteria harbouring plasmid pPM3, expressing a C-terminal His-Strep double tagged GpdC, or harbouring plasmid pPM2, expressing GpdC without any tag (Mock), were grown for 2 days at 37°C in the presence of 5% CO_2_ on sheep blood agar plates. Bacteria from 6 plates were scraped and lysed in 35 ml of 25 mM Tris-HCl, 150 mM NaCl, 0.2% triton, 1% NP-40%, 1% sodium deoxycholate, pH 7.6.

For His affinity purification, the lysates were clarified by centrifugation (10 min at 18500 g at RT) and the supernatant was diluted 1∶2 in PBS, 10 mM Imidazole, in the presence of proteinase inhibitor (cOmplete, Mini, EDTA-free Protease Inhibitor Cocktail Tablets, Roche). 3.5 ml of 50% slurry Chelating sepharose Fast Flow beads (GE Healthcare) was first coupled to Ni^2+^ according to the manufacturer instructions and then 1.75 ml of resin was added to the solution and incubated overnight at 4°C on a rotating wheel. The solution was then loaded into a column and the resin washed first with 25 column volumes (CV) of high salt buffer (50 mM Tris, 500 mM NaCl, pH 8) and then with 5 CV of low salt buffer (50 mM Tris, 100 mM NaCl, pH 8). Proteins were then eluted from the resin with 2 CV of elution buffer (50 mM Tris, 100 mM NaCl, 350 mM Imidazole, pH 8). The material eluted from the Ni^2+^ column was then diluted 1∶2 in PBS and 1 ml of 50% slurry (0.5 ml CV) *Strep*-Tactin Superflow resin (IBA, cat No: 2-1206-002) was added. The solution was then incubated overnight at 4°C on a rotating wheel. The solution was then loaded into a column and the flow through reloaded into the resin 2 more times. The resin was then washed 4 times with 10 CV of Buffer W (100 mM Tris, 150 mM NaCl, 1 mM EDTA, pH 8) and proteins eluted in 3 steps with 0.5 ml elution buffer (100 mM Tris, 150 mM NaCl, 1 mM EDTA, 2.5 mM desthiobiotin, pH 8). The proteins present in the elution fractions were identified by MS and immunoblotting, using anti-His for GpdC detection, anti-GpdG and anti-SiaC .

GpdC-sialidase co-purification was performed exactly as described above using *Cc5* Δ*PUL5* bacteria harbouring pPM3 plasmid or harbouring plasmid pPM2 (Mock). Proteins present in the elution fractions were identified by immunoblotting with anti-Strep antibodies to detect GpdC and anti-SiaC.

### *In vivo* radiolabeling with [^3^H] palmitate, immuno-precipitation and fluorography

Bacteria were inoculated to HeLa epithelial cells (ATCC CCL-2) in complete DMEM at 37°C with 5% CO_2_ at a moi of 20. 15–16 h post infection, [9,10-^3^H] palmitic acid (48 Ci/mmol; Perkin-Elmer Life Sciences) was added to a final concentration of 50 µCi/ml and incubation was continued for 8–9 h, by which time the bacterial culture had reached approximately 10^8^ bacteria/ml as described elsewhere [13]. Supernatants of 2×1 ml were collected without detaching epithelial cells from the wells. Bacteria corresponding to approximately 2×10^8^ cfu were then collected by centrifugation and pellets were combined from 2 ml and stored at −20°C until they were processed. Pellets were resuspended in 0.1 ml PBS TritonX 1% to lyze bacteria and sialidase was immuno-precipitated by addition of 10 µl rabbit polyclonal anti-SiaC for 1 h at RT on a rotating wheel. Protein A agarose slurry (Sigma) was then added in equal amounts for 30 min under constant rotation at RT. Samples were then centrifuged at 14000× g for 2 min at RT, supernatant was discarded and pellets were washed with 0.5 ml PBS 0.1% Triton which was repeated 4 times. Captured proteins were eluted by addition of 50 µl Lämmli buffer (1% SDS, 10% glycerol, 50 mM dithiothreitol, 0.02% bromophenol blue, 45 mM Tris, pH 6.8) for 5 min at 85°C. Samples were centrifuged again and supernatant was carefully separated from the agarose beads and loaded on SDS PAGE gels using 10% polyacrylamide. After gel electrophoresis, gels were fixed in 25∶65∶10 isopropanol∶water∶acetic acid overnight and subsequently soaked for 30 min in Amplify (Amersham). Gels were vacuum dried and exposed to SuperRX autoradiography film (Fuji) for 13days until desired signal strength was reached.
