# Correction: The *N*-glycan Glycoprotein Deglycosylation Complex (Gpd) from *Capnocytophaga canimorsus* Deglycosylates Human IgG

**DOI:** 10.1371/journal.ppat.1005352

**Published:** 2015-12-15

**Authors:** Francesco Renzi, Pablo Manfredi, Manuela Mally, Suzette Moes, Paul Jenö, Guy R. Cornelis

The authors would like to correct Fig 6B. The blot depicted in Fig 6B contains two errors: 1) the blot has been assembled by cropping and moving lanes from different parts of a same membrane without indication of this manipulation in the figure and in the legend; 2) the blot displays duplicated data in two lanes. These errors occurred during assembly of the final figure. The authors have corrected Fig 6B replacing the duplicated lanes with the correct ones and have boxed the lanes that were cropped and moved from the same or different blots.

**Fig 6:**
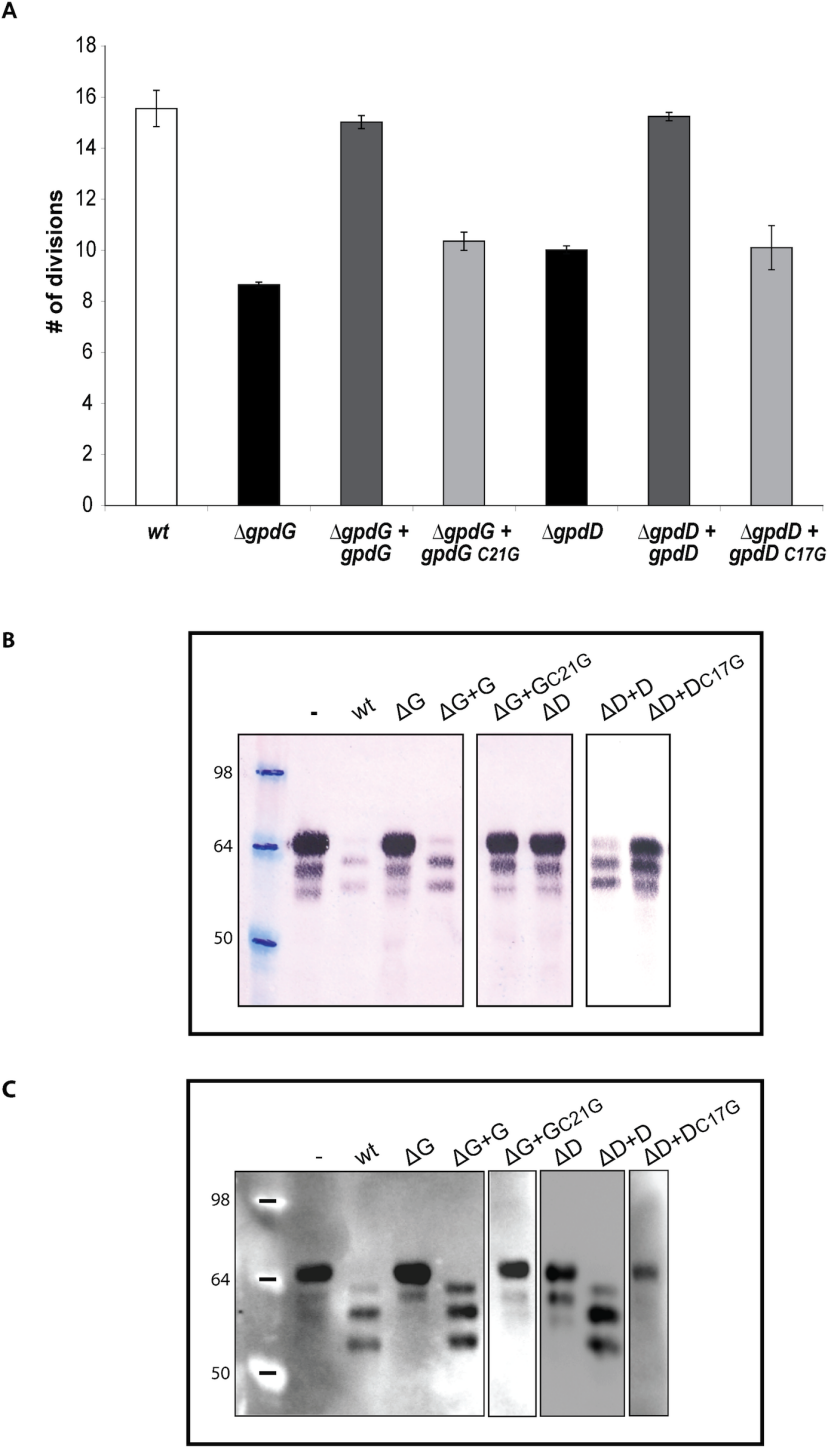
Lipid modification of GpdD and GpdG is essential for their activity. (A) Number of divisions after 23 h growth on HEK293 cells of *ΔgpdG* bacteria complemented with *gpdG*_*C21G*_ and of *ΔgpdD* bacteria complemented with *gpdD*_*C17G*_. (B) Fetuin glycosylation state of samples incubated for 2 hours in the presence of the different strains, determined by staining with SNA. (C) Same as B analyzed by western blot with anti-fetuin antibodies. The boxes indicate lanes that have been cropped and moved either from a same or a different blot.

The authors confirm that these changes do not alter their findings. The authors have provided raw, uncropped blots as Supporting Information.

## Supporting Information

S1 Fig Uncropped blots. Original unmodified blots used for the assembly of Fig 6B. The lanes used in Fig 6B are identified and labeled.
(TIF)
