# Supplementary figures and images for: Correction: The N-glycan Glycoprotein Deglycosylation Complex (Gpd) from Capnocytophaga canimorsus Deglycosylates Human IgG
Source: PLoS Pathog. 2015 Dec 15;11(12):e1005352. doi: 10.1371/journal.ppat.1005352 (PMC4679373; doi:10.1371/journal.ppat.1005352)

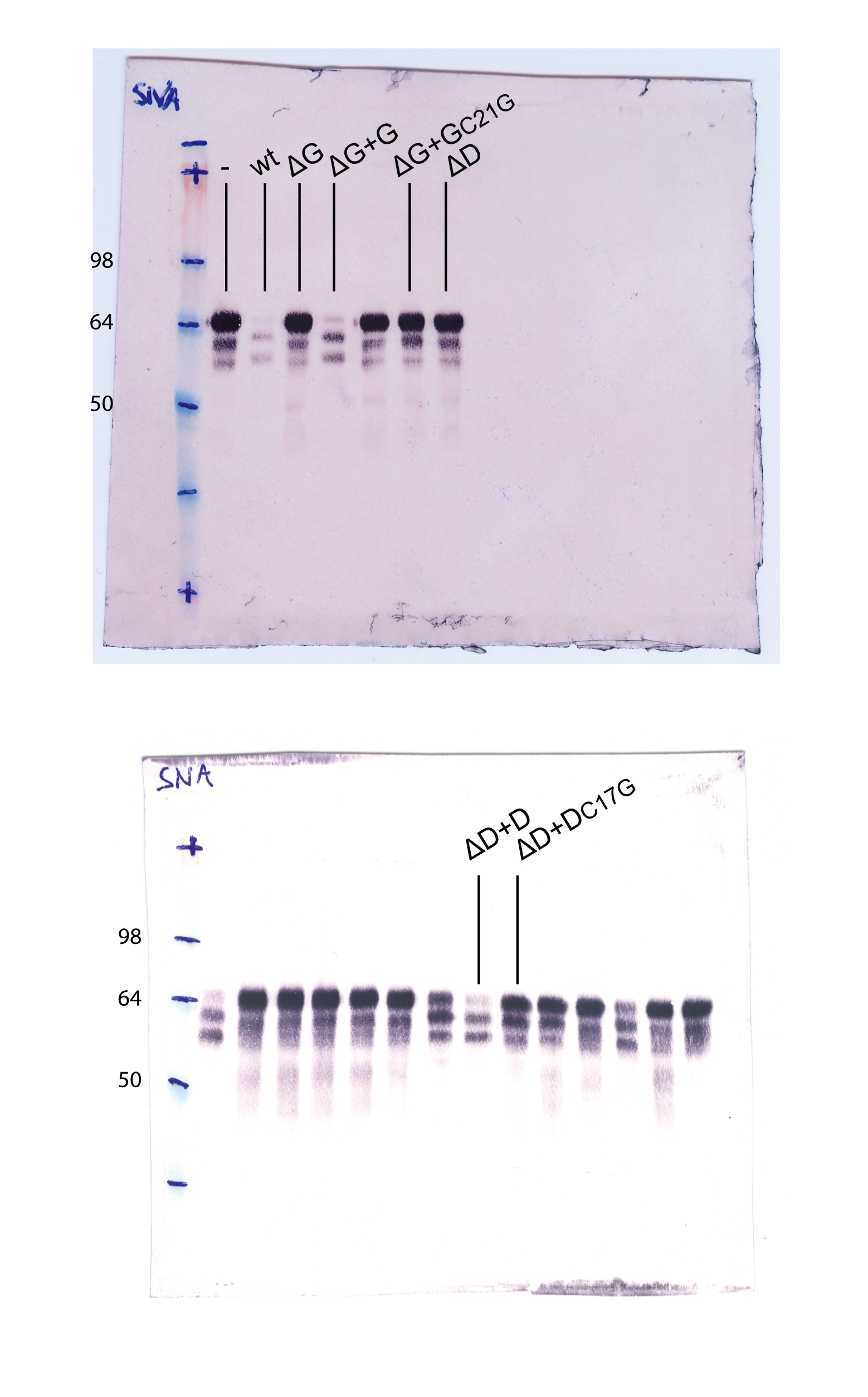

Supplement: S1 Fig — Original unmodified blots used for the assembly of Fig 6B. The lanes used in Fig 6B are identified and labeled. (TIF) [file ppat.1005352.s001.tif]
